# Genome-Wide Identification and Analysis of *Arabidopsis* Sodium Proton Antiporter (NHX) and Human Sodium Proton Exchanger (NHE) Homologs in *Sorghum bicolor*

**DOI:** 10.3390/genes9050236

**Published:** 2018-05-03

**Authors:** P. Hima Kumari, S. Anil Kumar, Katam Ramesh, Palakolanu Sudhakar Reddy, M. Nagaraju, A. Bhanu Prakash, Trushar Shah, Ashley Henderson, Rakesh K. Srivastava, G. Rajasheker, A. Chitikineni, Rajeev K. Varshney, P. Rathnagiri, M. Lakshmi Narasu, P. B. Kavi Kishor

**Affiliations:** 1 Department of Genetics, Osmania University, Hyderabad 500 007, India; phimakumari@gmail.com (P.H.K.); anilkumarou@gmail.com (S.A.K.); nagarajmarka@gmail.com (M.N.); g.rajashekharou@gmail.com (G.R.); 2 Centre for Biotechnology, Institute of Science & Technology, JNT University, Hyderabad 500 085, India; mangamoori@gmail.com; 3 Department of Biological Sciences, Florida A&M University, Tallahassee, FL 32307, USA; Ramesh.katam@famu.edu (K.R.); ashleylynnhen@gmail.com (A.H.); 4 Center of Excellence in Genomics & Systems Biology, International Crops Research Institute for the Semi-Arid Tropics (ICRISAT), Patancheru, Hyderabad 502 324, India; p.sudhakarreddy@cgiar.org (P.S.R.); a.bhanuprakash@cgiar.org (A.B.P.); r.k.srivastava@cgiar.org (R.K.S.); A.Gorantla@cgiar.org (A.C.); r.k.varshney@cgiar.org (R.K.V.); 5 IITA-Kenya c/o International Livestock Research Institute (ILRI), PO Box 30709, Nairobi 00100, Kenya; tm.shah@cgiar.org; 6 Ottawa University, Ottawa, KS 66067, USA; 7 Genomix CARL Pvt. Ltd. Rayalapuram Road, Pulivendula, 516 390, Kadapa, Andhra Pradesh, India; giripolava@gmail.com

**Keywords:** abiotic stress, amiloride, CBL-CIPK pathway, sodium-proton exchanger, sodium-proton antiporter, *Sorghum bicolor*

## Abstract

Na^+^ transporters play an important role during salt stress and development. The present study is aimed at genome-wide identification, in silico analysis of sodium-proton antiporter (NHX) and sodium-proton exchanger (NHE)-type transporters in *Sorghum bicolor* and their expression patterns under varied abiotic stress conditions. In *Sorghum*, seven *NHX* and nine *NHE* homologs were identified. Amiloride (a known inhibitor of Na^+^/H^+^ exchanger activity) binding motif was noticed in both types of the transporters. Chromosome 2 was found to be a hotspot region with five sodium transporters. Phylogenetic analysis inferred six ortholog and three paralog groups. To gain an insight into functional divergence of *SbNHX*/*NHE* transporters, real-time gene expression was performed under salt, drought, heat, and cold stresses in embryo, root, stem, and leaf tissues. Expression patterns revealed that both *SbNHXs* and *SbNHEs* are responsive either to single or multiple abiotic stresses. The predicted protein–protein interaction networks revealed that only SbNHX7 is involved in the calcineurin B-like proteins (CBL)- CBL interacting protein kinases (CIPK) pathway. The study provides insights into the functional divergence of *SbNHX*/*NHE* transporter genes with tissue specific expressions in *Sorghum* under different abiotic stress conditions.

## 1. Introduction

To cope with high salt concentrations in the soil and the toxic effects of Na^+^ and Cl^-^ ions, plants have evolved multiple mechanisms like exclusion of salt at the membrane level or inclusion of it in the vacuoles [1]. Ions that are sequestered into the vacuoles would be used by the plants as a cheap source of osmoticum that can help to absorb water [2]. Both ion and pH homeostasis play crucial roles in diverse cellular processes that control plant growth, development, and stress tolerance [3]. In general, the cytoplasmic pH value is determined to be above neutrality (pH 7.2–7.6), which is controlled by an array of buffering molecules like K^+^, Na^+^/K^+^, cation/proton exchangers like Ca^2+^/H^+^, sodium-proton antiporters (*NHX*), proton/nutrient-transporters and H^+^-translocating enzymes [4]. Na^+^ enters into the cell through two non-selective cation channels (NSCC): voltage-dependent and voltage-independent cation (VIC) channels. VIC channels are thought to be the major way for the movement of Na^+^ into plant cells [5,6]. NHX and sodium-proton exchanger (NHE) transporters belong to the cation proton antiporter1 CPA1 family [7,8]. It appears that CPA1 family has evolved from ancestral sodium-proton antiporter (*NhaP*) genes in prokaryotes [8,9,10]. Their primary physiological functions are regulation of cytoplasmic pH, extrusion of H^+^ generated during metabolism in exchange of Na^+^ or K^+^ ions into cytoplasm and vacuoles in plants and animals [11,12,13].

NHX1 was the first plant NHX identified in *Arabidopsis* and its overexpression lead to salt tolerance in plants [14,15]. Among the eight NHX members, NHX1 and NHX2 are the most abundant proteins in *Arabidopsis* [16]. In plants, NHX7 or salt overly sensitive (SOS1) and NHX8 are located in the plasma membrane and named PM-class [17]; NHX5 and NHX6 are intracellularly located in the endosomal compartments and named Endo-class [18,19]; NHX1 to NHX4 are in the tonoplast and named Vac-class [20]. NHX transporters are associated with salt tolerance, long-distance transport of Na^+^ from root to shoot, protein targeting and trafficking, and stomatal functioning [18,19,21,22,23,24]. Like NHX-type proteins, NHE-type protein family members are required for several key cellular processes like regulation of intracellular pH, cell volume and reabsorption of Na^+^ in the kidney and gastrointestinal tract [25]. The members of this family may have a similar structure, containing 10 to 12-transmembrane domains, large C-terminal domain and function as homodimers [26]. Bobulescua et al. [27] pointed out that NHE-type family of proteins fall into two subfamilies; the plasma membrane bound NHE 1-5, and the organellar membrane-bound NHE 6–9. The C-terminal domains may be cytosolic as in the case of NHE1 [28], or extracellularly exposed as in NHE3 [29]. It appears that *NHX* genes have evolved from plasma membrane NHE sequences [10]. However, functions of NHE members in plants are not known completely.

*Sorghum bicolor* is a moderately stress-tolerant crop suitable for dry land cultivation, ensuring productivity and access to food when other crops fail. *S. bicolor* is the fifth most important cereal crop and the second most important staple food grain in the semi-arid tropics. It is a self-pollinated diploid, C_4_ photosynthetic plant, which makes it adaptable to high temperature. It is an efficient biomass accumulator, provides feed, fodder and fuel, and shows genetic diversity with smaller genome [30]. Recently, NHX-like protein (NHXLP), which is plasma membrane bound and helps in Na^+^ exclusion, has been detected in *S. bicolor* [31]. This shows that *S. bicolor* may have NHXLP- and NHE-type genes in their genomes besides NHX for carrying out redundant functions. However, the tissue-specific expressions of *NHX* and *NHE*-type exchangers and their association with different abiotic stresses in *S. bicolor* are unknown. Earlier, genome-wide analysis of CPA1 family was carried out for Ca^2+^ transporters [32], K^+^ transporters [33,34], and NHX-type genes in poplar [33], but not for NHE homologs in plants. In this study, genome-wide scanning of *Arabidopsis NHX* and human *NHE* homologs in *Sorghum* was carried out including their chromosomal locations, gene structures, conserved motifs, phylogenetic relationship, transcript expressions in different tissues, promoter analysis, and predicted protein–protein interactions.

## 2. Materials and Methods

### 2.1. Plant Material and Abiotic Stress Treatments

*Sorghum* (*S. bicolor* L. BTx623) seeds collected from the International Crops Research Institute for the Semi-Arid Tropics (ICRISAT, Patancheru, India) were sown in earthen pots containing 4.5 kg of garden soil and manure (4:1) and maintained in green house at 28/20 °C day/night temperatures and relative humidity of 60±5% to 81±2%. Seventy-five-day-old *Sorghum* variety BTx623 plants were treated with salt (200 mM NaCl), drought (200 mM mannitol), heat (42 °C), and cold (4 °C) stresses for 4 h. Control (without any stress) plants were treated with tap water. Embryo, root, stem, and leaf tissue samples were collected immediately, snap frozen in liquid nitrogen and stored at −80 °C until RNA extraction.

### 2.2. Identification and Characterization of Sodium Transporters

All the 16 cDNA sequences of *Arabidopsis* and human Na^+^ transporters (NHX1-7, [35]; NHE1-9) were collected from NCBI database (http://www.ncbi.nlm.nih.gov/) (File S1) and blasted against the *S. bicolor* genome in the Gramene database by default settings. Different software tools like Genscan [36] for gene prediction, Gene Structure Display Server [37], for gene structure analysis, TMHMM [38], for topology analysis of transmembrane domains and WoLFPSORT [39] for subcellular localization of the predicted gene, NetPhos 3.1 [40] for phosphorylation sites, MEME [41] for conserved motifs with parameters like 20 number of motifs, 2–20 motif sites, 6–20 wide motif width were used. SMART program was employed to check for the presence of sodium proton exchanger domains [42]. The SbNHX and SbNHE proteins were modelled using I-TASSER tool [43].

### 2.3. Phylogeny, Divergence, Promoter Analysis, Physical Gene Mapping, and Co-Expression Analysis

Phylogenetic tree was constructed with amino acid sequences of *Sorghum bicolor* (Sb), *Populus trichocarpa* (Pt), *Arabidopsis thaliana* (At), *Eucalyptus grandis* (Eg), *Medicago truncatula* (Mt), *Vitis vinifera* (Vv), *Glycine max* (Gm), *Oryza sativa* (Os), *Brachypodium distachyon* (Bd), *Zea mays* (Zm), and *Physcomitrella patens* (Pp) using MEGA 6.0 software, by the Neighbor-Joining method [44]. The reliability of the phylogenetic tree was estimated using bootstrap values with 1000 replicates. Gene duplication events were also investigated using phylogenetic tree based on 70% similarity and 80% coverage of aligned sequences [45,46]. Synonymous (d_S_) and non-synonymous (d_N_) substitution rates were calculated using the PAL2NAL program [47]. In silico promoter analysis was carried out for 1 kb sequence upstream to all the *Sorghum* NHE/NHX transporters using PLACE [48] and PlantCARE [49] software. Physical mapping of the transporters was constructed based on their location on the *Sorghum* genome. The predicted protein–protein interaction (PPI) map of SbNHXs/NHEs was generated from the STRING database [50].

### 2.4. RNA Isolation and Quantitative Real-Time PCR Analysis

Total RNA was isolated from all the samples using Macherey-Nagel NucleoSpin RNA plant kit (740949.50) by following the instructions manual. DNaseI was used to eliminate genomic DNA contamination in RNA samples. The concentration and purity of RNA samples were checked using Eppendorf BioPhotometer. Total RNA (2 µg) was taken as template for first strand cDNA synthesis using RevertAid First Strand cDNA Synthesis Kit (#K1622, Thermo Scientific EU, Reinach, Switzerland). The relative expression levels of *SbNHX*/*NHE* genes were studied using 2X applied biosystems (ABI) Master Mix with gene specific primers (File S1). ABI 7500 real-time PCR system (Applied Biosystems, Foster City, CA, USA) was used with the following thermal cycling conditions of 95 °C for 5 min followed by 40 cycles of 95 °C for 30 s, 57 °C for 30 s, and 72 °C for 30 s. The expression of each *SbNHX*/*NHE* gene in various samples was normalized with *EIF4α* and *PP2A* reference genes [51]. The experiment was performed with two biological replicates and for each sample, three technical replicates were used. The specificity of the PCR reaction was confirmed by melting curve analysis of the amplicons. Comparative 2^−ΔΔCT^ method was used to calculate the relative quantities of each transcript in the samples [52].

## 3. Results

### 3.1. Identification and Characterization of NHX/NHE Transporters in Sorghum

**Figure 1:**
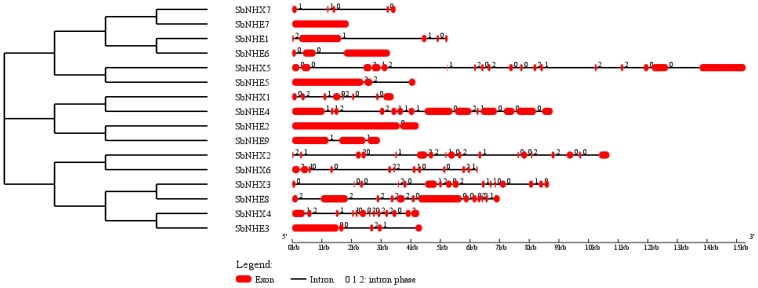
Gene characterization of sodium transporters. Exons are represented as red boxes and introns as black lines. Intron phases 0, 1, and 2 are indicated by the numbers 0, 1, and 2.

**Figure 2:**
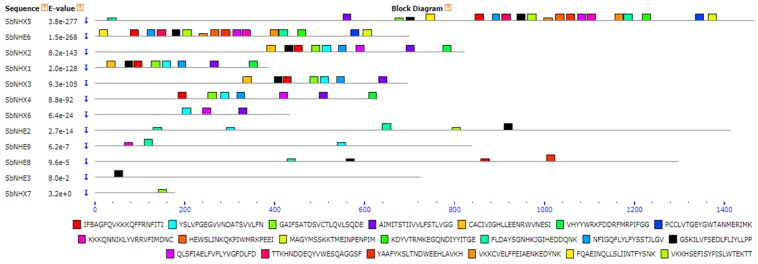
Conserved motif analysis of SbNHX/NHE proteins. Amiloride motif (LLFIYLLPPI) is represented in black. Sb: *Sorghum bicolor*; NHX: sodium proton antiporter; NHE: sodium proton exchanger.

**Figure 3:**
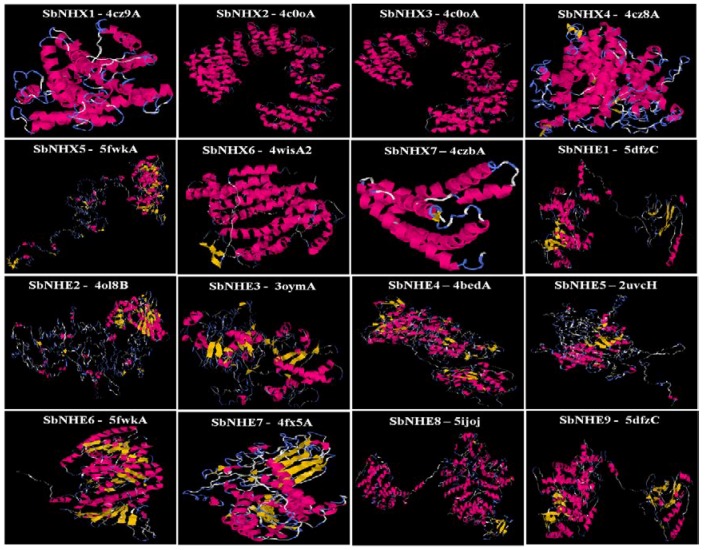
Structural analysis of seven SbNHX and nine SbNHE modeled proteins. The best Protein Data Bank (PDB) structural analog for each transporter is represented in the figure.

A total of 16 *SbNHX*/*NHE* transporter homologs (*Arabidopsis* NHX1-NHX7 and human NHE1-9) were identified in *S. bicolor* genome. The coding and amino acid sequence for each SbNHX/NHE transporter was predicted using Genscan tool (File S1). The number of exons vary from one (*SbNHE7*) to 18 (*SbNHX2* and *SbNHX5*) among the transporters (Figure 1; Table 1). The number of transmembrane domains for Na^+^ transporters varies from 0 to 8 (Table 1, Figure S1). The predicted proteins in *Sorghum* are localized on the plasma membrane, chloroplast, nucleus, endoplasmic reticulum, and mitochondria (Table 1). SbNHX/NHE are more phosphorylated with protein kinase C, protein kinase A (PKA), and cyclin-dependent protein kinase (CDC2) and very less with ataxia telangiectasia mutated (ATM). Serine has been found as the most common site for phosphorylation compared to threonine and tyrosine (Table 2). Motif prediction was carried out by MEME, and it showed from 0 to 20 motifs with a conservation and variation. No motifs were observed in SbNHE1, SbNHE4, SbNHE5, and SbNHE7. Amiloride drug binding site (LLFIYLLPPI), typical characteristic feature of NHX transporters was seen in SbNHX1, SbNHX2, SbNHX3, SbNHX5, SbNHE2, SbNHE3, SbNHE6, and SbNHE8, but absent in SbNHX4, SbNHX6, SbNHX7, and SbNHE9 (Figure 2). Sodium proton exchanger domain was observed in all the SbNHXs but not in SbNHEs. SbNHEs exhibit domains like ovate, reverse transcriptase (RVT), integrase core domain (RVE), transposase, PKinase, heat shock protein90 (HSP90), von Willebrand factor type A domain (VWA), domain of unknown function (DUF), and RVT1 (Table 1). All the SbNHX and SbNHE proteins were modeled using I-TASSER software to find out their 3D structures. For all the proteins, 3D structures were constructed based on the similar template obtained from Protein Data Bank (PDB). All the predicted SbNHX/NHE models constructed showed a C-Score range from −0.20 to −3.48, indicating the predicted proteins are constructed with high accuracy (Figure 3, File S1).

### 3.2. Phylogeny, Divergence, Promoter Analysis, and Physical Genome Mapping

**Figure 4:**
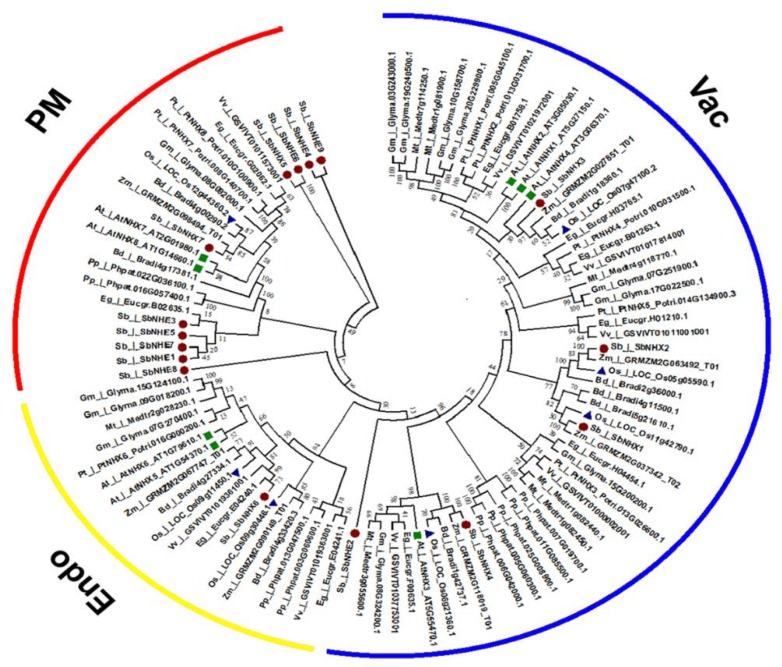
Phylogenetic tree of sodium transporters between *Sorghum bicolor* (Sb), *Populus trichocarpa* (Pt), *Arabidopsis thaliana* (At), *Eucalyptus grandis* (Eg), *Medicago truncatula* (Mt), *Vitis vinifera* (Vv), *Glycine max* (Gm), *Oryza sativa* (Os), *Brachypodium distachyon* (Bd), *Zea mays* (Zm), and *Physcomitrella patens* (Pp). Predicted amino acid sequences were used for construction of the tree. The tree was constructed by the Neighbor Joining method using MEGA version 6.0. Values indicate the number of times (as apercentage) that each branch topology was found during bootstrap analysis.

**Figure 5:**
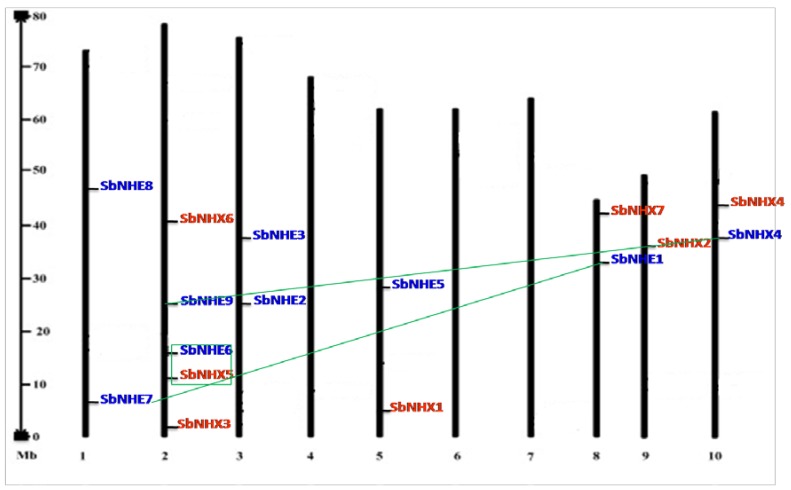
Physical mapping of *Sorghum NHX/NHE* genes. The three paralog gene pairs are represented with lines (*SbNHE1* and *SbNHE7*, *SbNHX4* and *SbNHE9*) and a box (*SbNHX5* and *SbNHE9*). All the *SbNHX* genes are represented in maroon and all *SbNHE* genes in blue.

The phylogenetic tree displayed three major clusters. Cluster 1 represented all the vacuolar membrane transporters including SbNHX1, SbNHX2, SbNHX3, SbNHX4, and SbNHE2. Cluster 2 formed all the endomembrane-located transporters like SbNHX6. Cluster 3, on the other hand, represented all the plasma membrane bound transporters such as SbNHX5, SbNHE1, SbNHE3, SbNHE4, SbNHE5, SbNHE6, SbNHE7, SbNHE8, and SbNHE9. Not surprisingly, similar to human NHE, all the *Sorghum* NHE homologs (except SbNHE2) clustered on the plasma membrane (Figure 4). Out of three paralog gene pairs, two segmental (*SbNHE1* and *SbNHE7*, *SbNHE4* and *SbNHE9*) and one regional (*SbNHX5* and *SbNHE6*) duplications were observed. *Sorghum* showed six ortholog pairs, four with maize (*SbNHX1* and Z*M2G037342*, *SbNHX2* and *Zm2G063492*, *SbNHX4* and *Zm2G118019*, *SbNHX7* and *Zm2G098494*) and two with *Eucalyptus* (*SbNHX6* and *Eucgr.E04240.1*, *SbNHE3* and *Eucgr.BO2635.1*) (Figure 4). Synonymous and non-synonymous substitution rates were calculated for paralog gene pairs. d_N_/d_S_ was found to be below <1, implying that the genes underwent a positive Darwinian selection or a purifying selection (Table 2). One kilobase (kb) sequence (File S1), upstream of the transcription start site was analyzed for *cis*-acting elements in all the *SbNHX*/*NHE* genes (Table 3). The identified *cis*-acting elements are grouped into hormone (ABA, auxin, gibberellic acid, ethylene, methyl jasmonic acid, and salicylic acid), stress (drought, heat shock, low temperature, salt, fungal, defense, and light), and other-responsive factors (guard cell specific (KST1), salt-responsive element (GT1) motif, O2 site, and zein metabolism regulation involved in circadian control). Special Protein 1 motif (SP1), stress-responsive and G BOX, positive regulators of senescence were found to be the highest number of *cis*-acting elements in SbNHX/NHE. GT1 and KST1 elements were noticed in all the promoter sequences of *SbNHX*/*NHE* genes (Table 3). In *Sorghum*, Na^+^ transporters were detected on seven out of 10 chromosomes, but no transporters were noticed on chromosomes 3, 4, and 6. Five genes are located on chromosome 2, three genes on chromosome 1, two genes on chromosomes 5, 8, and 9, and one gene each on chromosomes 7 and 10. Chromosome 2 has been found to be a hotspot region for SbNHX/NHE transporters (Figure 5, File S1).

### 3.3. Protein–Protein Interaction Analysis Prediction

**Figure 6:**
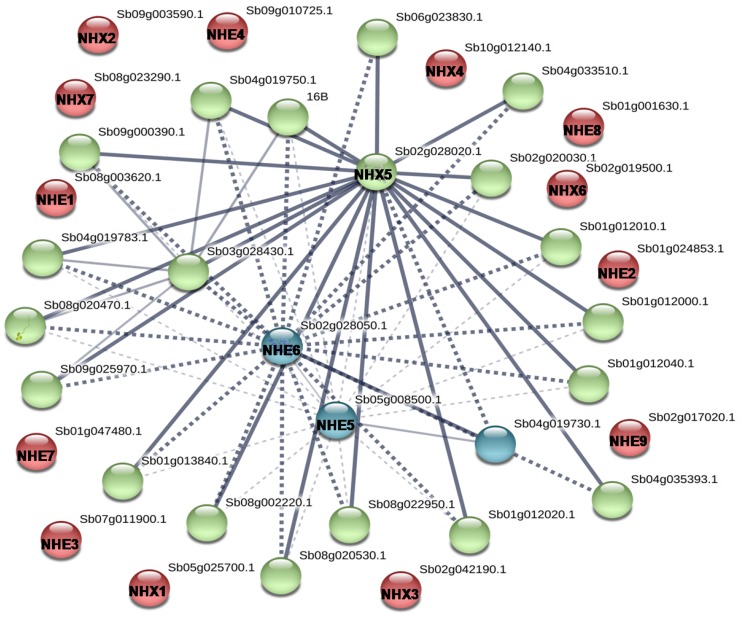
String analysis of SbNHX/NHE interacting proteins. All the SbNHX/NHE transporters are represented in bold.

In the predicted protein–protein interaction map, SbNHX/NHE displayed interactions with several other transporter proteins of *Sorghum*. SbNHX5 and SbNHE6 have been noticed as centers for interactions (Figure 6). When individual proteins (SbNHX and SbNHEs) were checked for their interaction, they showed interactions with various calcineurin B-like proteins (CBLs) like CBL02, CBL03, CBL05, and CBL08. SbNHX/NHE also showed interactions with various CBL-interacting protein kinases (CIPKs) like CIPK01, CIPK14, CIPK17, and CIPK22 (Figure S2).

### 3.4. Expression Analysis of SbNHX/NHE Transporters in Different Tissues Treated with Diverse Abiotic Stresses

**Figure 7:**
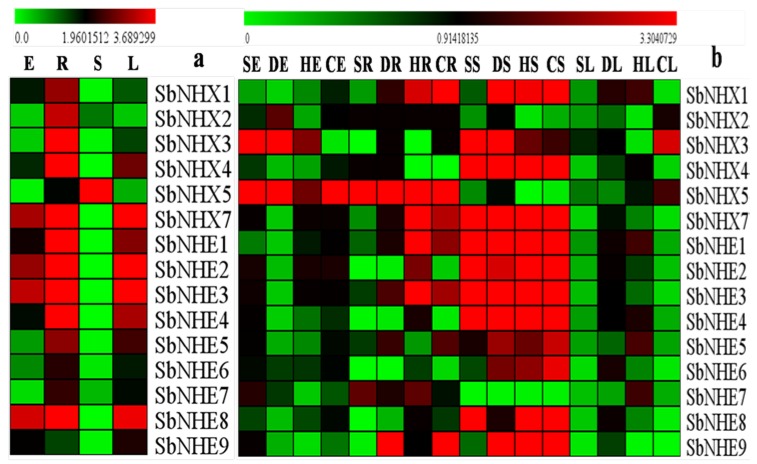
Relative expression analysis of *SbNHX*/*NHE* transporter genes in *Sorghum*. (**a**) Native expressions of the *SbNHX*/*NHE* transporters. (**b**) *SbNHX*/*NHE* transporters expressions to salt, drought, heat, and cold stresses. Relative expression of transporters is shown during different stress conditions in comparison to its corresponding controls. Values represent the expression levels obtained after normalizing against control tissues. All samples were analyzed in triplicate, in two independent experiments. Names on the horizontal axis indicate the tissues and the vertical axis represents various genes. E: embryo; R: root; S: stem; L: leaf; S: salt; D: drought; H: heat; C: cold. Each color represents the relative expression levels.

*SbNHX*/*NHE* transporters expression levels were analyzed in *Sorghum* embryos, roots, stems, and leaves exposed to salt, drought, heat, and cold stresses. *SbNHX*/*NHE* displayed differential gene expression levels among the four tissue types. Native expression of the transporters was high in roots followed by leaf, stem, and embryo, which may be involved in growth and developmental processes (Figure 7a). *SbNHX7* also recorded higher transcript abundance in all four tissues under native conditions (Figure 7a). Among the four stresses, high transcript levels were recorded in cold followed by salt, heat, and drought stresses. Among the genes, *SbNHX1* and *SbNHX4* showed higher expression in cold-treated stems, *SbNHX2* in drought-stressed embryos, *SbNHX3* in salt-exposed embryos, *SbNHX5* in salt-stressed roots, *SbNHX7* in drought-treated stems. Expression of *SbNHE1*, *SbNHE4*, *SbNHE5*, *SbNHE6*, and *SbNHE9* was high in cold-exposed stems, *SbNHE2*, *SbNHE3*, and *SbNHE8* in heat-treated stems, and *SbNHE7* in salt-stressed root tissues (Figure 7b).

## 4. Discussion

Salt stress limits plant growth and productivity due to ion toxicity and water uptake by decreasing the water potential [53]. Plants can either efflux Na^+^ ions or compartmentalize them into vacuoles through NHXs family members [54]. Both NHX/NHE members belong to the CPA1 family of transporters [7,55] and play crucial roles in providing energy, and cell expansion in plants [56].

### 4.1. Identification and Structural Analysis of *SbNHX/NHE* Genes

In the present study, a total of seven *SbNHX* and nine *SbNHE* transporters have been identified in the genome of *S. bicolor* (Table 1), like in other cereals such as *Oryza sativa* and *Zea mays*, which also contain seven *NHX* members in all [35]. Bioinformatics analysis revealed that NHX members in *S. bicolor* can be grouped into three classes based on their plasma (SbNHX5 and SbNHX7), vacuolar (SbNHX1, SbNHX2, SbNHX3, and SbNHX4), and endomembrane (SbNHX6) localizations. Both SbNHX5 and SbNHX7 are grouped under plasma membrane in *Sorghum*. But in *Arabidopsis*, AtNHX7 and AtNHX8 have been identified under the same category [22]. While Bassil et al. [18] noticed two endomembrane transporters (NHX5 and NHX6) in *A. thaliana*, only one (SbNHX6) is identified under this category in *Sorghum* in the present study (Figure 4). Plasma membrane-bound transporters (SOS1/NHX7 and NHX8) help in the exclusion of Na^+^ ions from cytoplasm [57,58]. Likewise, vacuolar bound NHX members segment Na^+^ ions, sugars, and secondary products under stress conditions [59]. Thus, NHX members located on both the plasma and the tonoplast play critical roles in efflux and compartmentalization respectively, and maintain Na^+^ ion homeostasis. On the other hand, endomembrane-located AtNHX5 and AtNHX6 have been found necessary for regulating protein processing, trafficking of cellular cargo, plant growth and development [1,18,19]. In *Sorghum*, SbNHX6 has been found on the endomembrane, but not NHX5 (Figure 4). Variation in the number of NHX family members was observed in diverse taxa like *Arabidopsis thaliana* (8), *Populus trichocarpa* (8), *Brachypodium distachyon* (9), *Eucalyptus grandis* (10), and *Glycine max* (12) [35]. SbNHX genes are well conserved similar to poplar NHX [35] during the course of evolution (Figure 4). Introns were noticed in all *SbNHXs*, like in poplar [35], and also in *SbNHE* members excepting *SbNHE7* (Figure 1). It is also observed that plasma membrane-bound NHX members have a long C-terminal cytosolic tail, which helps in interacting with other proteins [22]. Similar to poplar NHX [35], SbNHX/NHE proteins were constructed with high accuracy.

Like NHX, NHE family members also appear as the important determinants of salt stress tolerance and hence are of enormous importance in agriculture [60]. While nine eukaryotic NHE members are present in humans [8], no information is available on the number of NHE transporters present in the genomes of higher plants and their subcellular localizations. In humans, NHE1-5 are located on plasma membrane, while NHE6-9 act as intracellular transporters [27]. All the SbNHEs are classified as plasma membrane bound except SbNHE2, which is predicted as a vacuolar membrane transporter (Figure 4). Contrary to the above report, the human isoforms HsNHE6 and HsNHE7 have been recognized as endosome-localized transporters [61,62].

### 4.2. Motif Identification, Promoter Analysis, Phylogenetic Tree, and Divergence

The N-termini of these proteins are comprised of a conserved domain LLFIYLLPPI, typical characteristic feature of NHX transporters in plants [63,64]. Amiloride binding site was found in all the NHX members of *Arabidopsis*, poplar [35], and in SbNHX1, SbNHX2, SbNHX3, SbNHX5, SbNHE2, SbNHE3, SbNHE6, and SbNHE8. Quiet surprisingly, it could not be detected in SbNHX4, SbNHX6, SbNHE7, and SbNHE9 (Figure 2; Table 1). All *SbNHX* and *SbNHE* promoter regions showed hormone, stress, and development-responsive *cis*-acting elements, indicating that these genes are regulated not only by abiotic stresses, but also by different hormones (Table 3). Similar to poplar NHX elements [35], abscisic acid-responsive elements (ABRE), auxin-responsive, fungal-responsive, circadian, low temperature-responsive (LTR), and heat shock elements (HSE) were noticed in *SbNHX*/*NHE* sequences. This infers that even NHE homologues are regulated by phytohormones and help perhaps in stress alleviation. Promoters of all SbNHX and SbNHE members showed GT1 and KST1 *cis*-elements. Plesch et al. [65] pointed out that KST1 is a guard cell-specific expression element that controls stomatal carbon dioxide uptake and water loss in crop plants during stress. Surprisingly, Sp1, a zinc finger transcription factor has also been detected in the promoter region of SbNHX/NHE members. Sp1 regulates the expression of many genes involved in various cellular functions such as differentiation, proliferation and apoptosis in cancer cells [66]. The presence of Sp1 transcription factor in *Sorghum* indicates that *SbNHX/SbNHE* group of genes may be associated with important cellular functions. G-box elements that act as positive regulators of early leaf senescence in rice [67] were detected in the promoter regions of *SbNHX/SbNHE*, implying that these genes also modulate leaf senescence. A phylogenetic tree indicated that SbNHX/NHE members fall into three paralog and six ortholog groups. In addition to rice (the common monocot ancestor), four ortholog *Sorghum* transporter events were observed with maize. Interestingly, *SbNHX6* and *SbNHE3* showed ortholog events with *Eucalyptus*. For paralog gene pairs, <1 substitution rates indicated that the genes underwent a positive Darwinian selection or a purifying selection [68].

### 4.3. Gene Expressions in Different Tissues under Abiotic Stresses

The *Sorghum* variety BTx623 is moderately tolerant to drought and adapted to high temperature but highly sensitive to cold. Genome-wide analysis for the identification and gene expressions of NHX and NHE members was carried out in this variety due to the availability and accessibility of its genome sequence [30]. Such an analysis helps to find out the role of NHX and NHE members in abiotic stress tolerance. Accordingly, in *Sorghum*, *NHX* and *NHE* genes were expressed in a tissue specific manner under different abiotic stress conditions. Among four tissues, noticeably high transcript levels were recorded in stems followed by roots, and embryos. *SbNHX5* displayed higher transcript levels in root and embryo tissues. Bassil et al. [18] pointed out that *NHX5* is required for plant growth and development. High transcript levels of *SbNHX7* under drought stress may be due to the presence of KST1, which controls stomatal carbon dioxide uptake and water loss in crop plants [65]. In spite of low number or absence of low temperature-responsive elements, *SbNHX1*, *SbNHX4*, *SbNHE1*, *SbNHE4*, *SbNHE5*, *SbNHE6*, and *SbNHE9* showed high transcript levels under cold stress conditions indicating a key role for them in temperature stress. The function of NHE homologues in plants is not yet known. For the first time, SbNHE transcript profile was carried out in *Sorghum* and found to be associated with abiotic stresses. Most of the *SbNHEs* displayed high expression in stem tissues under different abiotic stresses indicating their role in specific tissues during stress. Such tissue-specific expression patterns of *SbHSFs* were observed earlier in many plants including *S. bicolor* [69] and specifically *NHX* genes in poplar [35] and *NHXLP* gene in tomato [31]. These results indicate the involvement of NHX family members not only in salt stress, but also in different developmental processes like flower development and reproduction as also pointed out by Bassil et al. [18].

### 4.4. Protein–Protein Interaction Predictions

Our study revealed that all the SbNHX and SbNHE members, especially SbNHX5 and SbNHE6, interacted with many other proteins. The *Arabidopsis* AtNHX5 and AtNHX6 play a key role in plant development, and these genes have been used to improve salt tolerance in a variety of species [70]. Katiyar-Agarwal et al. [71] and Quintero et al. [72] revealed that C-terminal tails of NHX1 and SOS1 are essential for mediation of protein–protein interactions and ion selectivity. Wu et al. [73] also pointed out that hydrophilic C-terminus of *Salicornia europaea* vacuolar Na^+^*/*H^+^ antiporter is necessary for interaction and its function. It is known that CBL proteins interact and modulate the CIPK. In turn, kinases mediate the calcium signal transduction. SOS1/NHX7 is regulated by CBL and CIPK-mediated Ca^2+^ signaling pathway during Na^+^ expulsion [74]. In the present study, only SbNHX7 interacted with CBL5 and CIPK24. SOS3 or CBL4 is a Ca^2+^ sensor and escorts SOS2 to SOS1 to interact and activate it for Na^+^ efflux from the cell [75]. Thus, binding of CBL4 with Ca^2+^ is needed for its function during salt stress [76]. Ma et al. [77] found that overexpression of CPA1 or SOS1 increased the salt tolerance. This is also consistent with the present observation that SbSOS1/SbNHX7 is bound to the CBL5 and CIPK24. Unlike SbNHX7, other SbNHX members were bound only to the members of CBL, except NHX5, but not to CIPK24. Our findings thus revealed that SbNHX members interact with CBL members and play distinct roles during salt stress and plant developmental processes. Contrary to this, SbNHE members have not been found to interact with any one of the CBL members indicating such an interaction may not be necessary. However, some functional redundancy among NHX and NHE members cannot be ruled out in plants since both the groups are associated with abiotic stresses.

## 5. Conclusions

Genome-wide screening of *S. bicolor* revealed the presence of seven *SbNHX* and nine *SbNHE* homologs. While the NHX members fall into three subfamilies (plasma, vacuolar, and endomembranes), NHE family members form only two subfamilies (plasma and vacuolar). Interestingly, promoter regions showed GT1 and KST1 *cis*-elements, revealing their role in salt stress responsiveness and guard cell functioning respectively. Tissue-specific expression studies of *SbNHX* and *SbNHE* genes under abiotic stress conditions showed that they are associated with growth and developmental processes. This information would be useful in selecting candidate genes for functional validation in relation to abiotic stress tolerance during various developmental phases in crop species.

## Figures and Tables

**Table 1:** Characterization of *S. bicolor* NHX and NHE transporters.

ACC Number	Gene Name	No. ofAa	Chr	Domain	DBD	pI/MW	GRAVY	TMHMM	No. of Exons	Localization	Instability INDEX
Sb05g025700.1	*SbNHX1*	386	5	NHX	23–283	9.28/42,070.0	0.445	7	8	PM	40.26
Sb09g003590.1	*SbNHX2*	823	9	NHX	377–719	9.56/89,834.3	0.144	8	18	Chlo	48.13
Sb02g042190.1	*SbNHX3*	696	2	NHX	405–560	9.42/76,720.9	0.151	4	16	PM	44.18
Sb10g012140.1	*SbNHX4*	631	10	NHX	192–516	10.19/70,077.0	−0.009	8	13	PM	42.92
Sb02g028020.1	*SbNHX5*	1466	2	NHX	459–577	6.74/164,308.4	−0.483	2	18	Chlo	45.69
Sb02g019500.1	*SbNHX6*	434	2	NHX	191–346	6.41/48,771.7	−0.400	0	12	ER	47.89
Sb08g023290.1	*SbNHX7*	178	8	NHX	14–178	6.27/18,648.0	0.688	5	5	Cyto	24.22
Sb08g003620.1	*SbNHE1*	597	8	Ovate	319–375	5.25/62,189.4	−0.357	0	5	Chlo	46.11
Unknown	*SbNHE2*	1414	3	RVT	595–752	6.19/163,691.7	−0.361	0	2	Cyto	48.12
Unknown	*SbNHE3*	726	3	RVE	258–367	9.21/84,058.5	−0.587	0	5	Nucl	57.40
Unknown	*SbNHE4*	1826	10	Transposase	686–867	6.75/203,438.2	−0.738	0	14	Nucl	58.73
Sb05g008500.1	*SbNHE5*	955	5	PKinase	496–709	8.71/101,934.8	−0.006	2	3	PM	49.28
Sb02g028020.1	*SbNHE6*	699	2	HSP90	185–695	4.97/80,238.0	−0.609	0	3	Nucl	36.89
Sb01g047480.1	*SbNHE7*	637	1	VWA	169–355	6.07/67,727.9	−0.285	0	1	Chlo	50.99
Sb01g001630.1	*SbNHE8*	1298	1	DUF	48–362	6.94/145,110.4	−0.335	0	13	Cyto	39.59
Sb02g017020.1	*SbNHE9*	840	2	RVT1	65–214	9.10/95,836.4	−0.320	0	3	Mito	36.69

Aa: amino acid length; chr: chromosomal location; DBD: DNA binding domain; pI/MW: isoelectric point/molecular weight; TMHMM: transmembrame domain; NHX: sodium proton domain; HSP9: heat shock protein9; VWA: von Willebrand A; DUF: domain of unknown function; RVT: reverse transcriptase; PM: plasma membrane; Chlo: chloroplast; ER: endoplasmic reticulum; Cyto: cytoplasm; Nucl: nucleus; Mito: mitochondria; Sb: *Sorghum bicolor*; NHX: sodium proton antiporter; NHE: sodium proton exchanger.

**Table 2:** Divergence time of *Sorghum* paralog genes.

Gene 1	Gene 2	*d*_N_	*d*_S_	*d*_N_/*d*_S_
*SbNHX5*	*SbNHE6*	0.0047	0.3627	0.0130
*SbNHE1*	*SbNHE7*	1.7237	60.2714	0.0286
*SbNHE4*	*SbNHE9*	1.3993	50.2271	0.0279

*d*_S_, synonymous substitution; *d*_N_, non-synonymous substitution. Sb: *Sorghum bicolor*; NHX: sodium proton antiporter; NHE: sodium proton exchanger.

**Table 3:** *Cis*-acting element analysis of *SbNHX* and *SbNHE* gene promoters.

Gene	Hormone Responsive						Stress Responsive								Others								
	ABRE	TGAAACGAC	GARETCTGTTG	EREATTTCAAA	Me-Ja	TCACAGAAAAGGA	DRE	HSE	LTR	GT1	MYB	Box	TC rich	Sp1 motif	GARETCTGTTG	KST1	SKN1	Box4	Box I	G Box	II Box	O2 site	Circadian
*SbNHX1*	10	1	0	0	4	1	2	3	0	2	2	1	0	18	0	4	2	1	1	12	0	1	2
*SbNHX2*	2	3	1	1	7	2	0	3	0	5	2	2	2	3	1	8	6	3	4	7	0	1	1
*SbNHX3*	7	0	1	1	3	0	0	1	0	7	3	2	1	1	1	13	7	3	3	18	0	2	1
*SbNHX4*	3	1	0	0	4	0	1	2	0	4	4	1	4	1	0	8	6	3	1	8	2	0	2
*SbNHX5*	0	0	1	0	0	2	0	0	1	2	1	1	3	0	1	4	6	0	2	5	0	1	0
*SbNHX6*	0	1	5	2	2	1	0	1	0	4	5	2	5	2	5	13	3	2	4	3	1	2	3
*SbNHX7*	0	0	0	0	4	3	1	0	0	2	0	0	0	1	0	6	2	5	2	8	1	1	3
*SbNHE1*	4	0	1	1	4	1	3	1	2	8	0	0	2	2	1	4	5	1	1	9	0	1	1
*SbNHE2*	0	0	0	0	1	0	4	0	0	2	1	0	1	9	0	6	0	2	1	6	4	1	3
*SbNHE3*	1	1	0	0	1	3	0	0	1	4	6	0	0	2	0	5	7	0	0	4	3	0	3
*SbNHE4*	1	0	0	0	2	1	2	0	0	1	1	0	2	3	0	7	7	2	0	2	1	2	4
*SbNHE5*	1	0	0	0	5	0	3	5	0	4	3	3	1	4	0	8	2	2	0	3	1	1	2
*SbNHE6*	1	2	1	0	0	2	0	3	0	4	3	1	2	1	1	7	10	3	1	0	4	3	4
*SbNHE7*	0	0	2	0	2	3	0	2	1	2	2	1	2	3	2	6	3	1	3	5	0	1	4
*SbNHE8*	2	2	0	0	2	1	1	1	1	4	2	0	0	6	0	3	4	1	0	3	4	1	1
*SbNHE9*	5	1	1	0	12	0	1	1	0	2	4	2	0	5	1	4	5	0	0	10	2	1	8

ABRE: Abscisic acid-responsive elements; DRE (CACGTG): Drought-responsive (ACCGAC); HSE: heat shock-responsive elements(AGAAAATTCG); LTR: Low temperature-responsive (CCGAAA); GT1GMSCAM4 (*GAAAAA*): SALT-responsive; MYB (*WAACCA*/*YAACKG*/*CNGTTR*): drought-responsive; TGAACGAC; auxin-responsive: GCN4CAAGCCA; endosperm-responsive: GARETCTGTTG; gibberellic acid-responsive: EREATTTCAAA; ethylene-responsive: CGTCA; methyl jasmonic acid-responsive (CGTCA): Box-W1TTGAC; fungal-responsive: TCACAGAAAAGGA; salicylic acid-responsive: TC rich (ATTTTCTCCA); defense and stress-responsive: *KST1* (*TAAAG*); guard cell specific: *SKN1* (GTCAT); endosperm-responsive: Box 4 (ATTAAT): Box I (TTTCAAA): G Box (CACGTC): GT1 motif (GGTTAA): I Box (GATAAGGTG): and Sp1 motif (CC(G/A)CCC); light-responsive; O2 site: (GATGACATGA); Zein metabolism regulation: circadian (CAANNNNATC): involved in circadian control.

## Supplementary Materials

The following are available online at http://www.mdpi.com/2073-4425/9/5/236/s1, Figure S1: Transmembrane domains for SbNHX/NHE proteins, Figure S2: String analysis for individual *SbNHX*/*NHE* proteins, File S1: List of Sodium transporters, comparative list, phosphorylation sites, Cds, amino acids, promoters, phylogeny sequences, protein modeling, and primer sequences.

## References

[1] Bassil E., Coku A., Blumwald E. (2012). Cellular ion homeostasis: Emerging roles of intracellular NHX Na^+^/H^+^antiporters in plant growth and development. J. Exp. Bot..

[2] Bohnert H.J., Nelsen D.E., Jensen R.G. (1995). Adaptation to environmental stresses. Plant Cell.

[3] An R., Chen Q.J., Chai M.F., Lu P.L., Su Z., Qin Z.X., Chen J., Wang X.C. (2007). AtNHX8, a member of the monovalent cation: Proton antiporter-1 family in *Arabidopsis thaliana*, encodes a putative Li^+^/H^+^ antiporter. Plant J..

[4] Mojca B., Tanja B., Ljerka L., Nada K. (2009). A comparative genomic analysis of calcium and proton signaling/homeostasis in *Aspergillus* species. Fungal Genet. Biol..

[5] Amtmann A., Sanders D. (1999). Mechanisms of Na^+^ uptake by plant cells. Adv. Bot. Res..

[6] Schachtman D., Liu W. (1999). Molecular pieces to the puzzle of the interaction between potassium and sodium uptake in plants. Trends Plant. Sci..

[7] Maser P., Thomine S., Schroeder J.I., Ward J.M., Hirschi K., Sze H., Talke I.N., Amtmann A., Maathuis F.J., Sanders D. (2001). Phylogenetic relationships within cation transporter families of *Arabidopsis*. Plant Physiol..

[8] Brett C.L., Donowitz M., Rao R., Christopher L. (2005). Evolutionary origins of eukaryotic Na^+^/H^+^ exchangers. Am. J. Physiol. Cell Physiol..

[9] Xiang M., Feng M., Muend S., Rao R. (2007). A human Na^+^/H^+^ antiporter sharing evolutionary origins with bacterial NhaA may be a candidate gene for essential hypertension. Proc. Nat. Acad. Sci. USA.

[10] Rodríguez-Rosales P.M., Gálvez F.J., Huertas R., Aranda M.N., Baghour M., Cagnac O., Venema K. (2009). Plant NHX cation/protonantiporters. Plant Signal. Behav..

[11] Waditee R., Hibino T., Nakamura T., Incharoensakdi A., Takabe T. (2002). Overexpression of a Na^+^/H^+^ antiporter confers salt tolerance on a freshwater cyanobacterium, making it capable of growth in sea water. Proc. Nat. Acad. Sci. USA.

[12] Putney L.K., Denker S.P., Barber D.L. (2002). The changing face of the Na^+^/H^+^ exchanger, NHE1: Structure, regulation, and cellular actions. Ann. Rev. Pharmacol. Toxicol..

[13] Leidi E.O., Barragan V., Rubio L., El-Hamdaoui A., Ruiz M.T., Cubero B., Fernández J.A., Bressan R.A., Hasegawa P.M., Quintero F.J. (2010). The AtNHX1 exchanger mediates potassium compartmentation in vacuoles of transgenic tomato. Plant J..

[14] Apse M.P., Aharon G.S., Snedden W.A., Blumwald E. (1999). Salt tolerance conferred by overexpression of a vacuolar Na^+^/H^+^antiport in *Arabidopsis*. Science.

[15] Gaxiola R.A., Rao R., Sherman A., Grisafi P., Alper S.L., Fink G.R. (1999). The *Arabidopsis thaliana* proton transporters, AtNhx1 and Avp1, can function in cation detoxification in yeast. Proc. Natl. Acad. Sci. USA.

[16] Yokoi S., Quintero F.J., Cubero B., Ruiz M.T., Bressan R.A., Hasegawa P.M., Pardo J.M. (2002). Differential expression and function of *Arabidopsis thaliana* NHX Na^+^/H^+^ antiporters in the salt stress response. Plant J..

[17] Shi H., Ishitani M., Kim C., Zhu J.K. (2000). The *Arabidopsis thaliana* salt tolerance gene SOS1 encodes a putative Na^+^/H^+^ antiporter. Proc. Natl. Acad. Sci. USA.

[18] Bassil E., Ohto M.A., Esumi T., Tajima H., Zhu Z., Cagnac O., Belmonte M., Peleg Z., Yamaguchi T., Blumwald E. (2011). The *Arabidopsis* intracellular Na^+^/H^+^ antiporters NHX5 and NHX6 are endosome associated and necessary for plant growth and development. Plant Cell.

[19] Bassil E., Tajima H., Liang Y.C., Ohto M.A., Ushijima K., Nakano R., Esumi T., Coku A., Belmonte M., Blumwald E. (2011). The *Arabidopsis* Na^+^/H^+^antiporters NHX1 and NHX2 control vacuolar pH and K^+^ homeostasis to regulate growth, flower development, and reproduction. Plant Cell.

[20] Apse M.P., Blumwald E. (2007). Na^+^ transport in plants. FEBS Lett..

[21] Zhang H.X., Blumwald E. (2001). Transgenic salt-tolerant tomato plants accumulate salt in foliage but not in fruit. Nat. Biotechnol..

[22] Shi H., Quintero F.J., Pardo J.M., Zhu J.K. (2002). The putative plasma membrane Na^+^/H^+^ antiporter SOS1 controls long-distance Na^+^ transport in plants. Plant Cell.

[23] Barragan V., Leidi E.O., Andres Z., Rubio L., Luca A.D., Fernandez J.A., Cubero B., Pardo J.M. (2012). Ion exchangers NHX1 and NHX2 mediate active potassium uptake into vacuoles to regulate cell turgor and stomatal function in *Arabidopsis*. Plant Cell.

[24] Wu X.X., Li J., Wu X.D., Liu Q., Wang Z.K., Liu S.S., Li S.N., Ma Y.L., Sun J., Zhao L. (2016). Ectopic expression of *Arabidopsis thaliana* Na^+^(K^+^)/H^+^ antiporter gene, AtNHX5, enhances soybean salt tolerance. Genet. Mol. Res..

[25] Counillon L., Pouyssegur J. (2000). The expanding family of eukaryotic Na^+^/H^+^ exchangers. J. Biol. Chem..

[26] Orlowski J., Grinstein S. (1997). Na^+^/H^+^ exchangers in mammalian cells. J. Biol. Chem..

[27] Bobulescua I.A., Soleb F.D., Moe O.W. (2005). Na^+^/H^+^ exchangers: Physiology and link to hypertension and organ ischemia. Curr. Opin. Nephrol. Hypertense.

[28] Wakabayashi S., Pang T., Su X., Shigekawa M. (2000). A novel topology model of the human Na^+^/H^+^ exchanger isoform. J. Biol. Chem..

[29] Biemesderfer D., Reilly R.F., Exner M., Igarashi P., Aronson P.S. (1992). Immunocytochemical characterization of Na^+^-H^+^ exchanger isoform NHE-1 in rabbit kidney. Am. J. Physiol. Ren. Fluid Electrolyte Physiol..

[30] Paterson A.H., Bowers J.E., Bruggmann R., Dubchak I., Grimwood J., Gundlach H., Haberer G., Hellsten U., Mitros T., Poliakov A. (2009). The *Sorghum bicolor* genome and the diversification of grasses. Nature.

[31] Kumari P.H., Kumar A.S., Sivan P., Katam R., Suravajhala P., Rao K.S., Varshney R.K., Kishor P.B.K. (2017). Overexpression of a plasma membrane Na^+^/H^+^-antiporter-like protein (SbNHXLP) confers salt tolerance and improves fruit yield in tomato by maintaining ion homeostasis. Front. Plant Sci..

[32] Anshita G., Gohar T., Dinesh P., Sanjay G., Anil K. (2011). Genome-wide comparative in silico analysis of calcium transporters of rice and sorghum. Genom. Proteom. Bioinform..

[33] Amrutha R.N., Sekhar P.N., Varshney R.K., Kishor P.B.K. (2007). Genome-wide analysis and identification of genes related to potassium transporter families in rice (*Oryza sativa* L.). Plant Sci..

[34] Zhang Z., Zhang J., Chen Y., Li R., Wang H., Wei J. (2012). Genome-wide analysis and identification of HAK potassium transporter gene family in maize (*Zea mays* L.). Mol. Biol. Rep..

[35] Tian F., Chang E., Li Y., Sun P., Hu J., Zhang J. (2017). Expression and integrated network analyses revealed functional divergence of NHX-type Na^+^/H^+^ exchanger genes in poplar. Sci. Rep..

[36] Burge C.B., Karlin S. (1998). Finding the genes in genomic DNA. Curr. Opin. Struct. Biol..

[37] Guo A.Y., Zhu Q.H., Chen X., Luo J.C., Chuan Y.I. (2007). GSDS a gene structure display server. Yi Chuan.

[38] Moller S., Croning M.D.R., Apweller R. (2001). Evaluation of methods for the prediction of membrane spanning regions. Bioinformatics.

[39] Horton P., Park K.J., Obayashi T., Fujita N., Harada H.C.J., Nekai A.C.K. (2007). WoLF PSORT: Protein localization predictor. Nucleic Acids Res..

[40] Blom N., Gammeltoft S., Brunak S. (1999). Sequence-and structure-based prediction of eukaryotic protein phosphorylation sites. J. Mol. Biol..

[41] Bailey T.L., Gribskov M. (1998). Combining evidence using *p*-values: Application to sequence homology searches. Bioinformatics.

[42] Letunic I., Copley R.R., Schmidt S., Ciccarelli F.D., Doerks T., Schultz J., Ponting C.P., Bork P. (2004). SMART 4.0: Towards genomic data integration. Nucleic Acids Res..

[43] Yang J., Yan R., Roy A., Xu D., Poisson J., Zhang Y. (2015). The I-TASSER Suite: Protein structure and function prediction. Nat. Methods.

[44] Tamura K., Stecher G., Peterson D., Filipski A., Kumar S. (2013). MEGA6: Molecular Evolutionary Genetics Analysis Version 6.0. Mol. Biol. Evol..

[45] Gu Z., Cavalcanti A., Chen F.C., Bouman P., Li W.H. (2002). Extent of gene duplication in the genomes of *Drosophila*, nematode, and yeast. Mol. Biol. Evol..

[46] Yang S., Zhang X., Yue J.X., Tian D., Chen J.Q. (2008). Recent duplications dominate NBS-encoding gene expansion in two woody species. Mol. Genet. Genom..

[47] Suyama M., Torrents D., Bork P. (2006). PAL2NAL: Robust conversion of protein sequence alignments into the corresponding codon alignments. Nucleic Acids Res..

[48] Higo K., Ugawa Y., Iwamoto M., Korenaga T. (1999). Plant *cis*-acting regulatory DNA elements (PLACE) database. NucleicAcids Res..

[49] Lescot M.P., Dehais G., Thijs K., Marchal Y., Moreau Y., Van de Peer Y., Rouzé P., Rombauts S. (2002). PlantCARE, a database of plant *cis*-acting regulatory elements and a portal to tools for in silico analysis of promoter sequences. Nucleic Acids Res..

[50] Szklarczyk D., Franceschini A., Kuhn M., Simonovic M., Roth A., Minguez P., Doerks T., Stark M., Muller J., Bork P. (2011). The STRING database in 2011: Functional interaction networks of proteins, globally integrated and scored. Nucleic Acids Res..

[51] Reddy P.S., Reddy D.S., Sivasakthi K., Bhatnagar-Mathur P., Vadez V., Sharma K.K. (2016). Evaluation of sorghum (*Sorghum bicolor* (L.)) reference genes in various tissues and under abiotic stress conditions for quantitative real-time PCR data normalization. Front. Plant Sci..

[52] Schmittgen T.D., Livak K.J. (2008). Analyzing real-time PCR data by the comparative CT method. Nat. Protoc..

[53] Munns M., Tester M. (2008). Mechanisms of salinity tolerance. Ann. Rev. Plant. Biol..

[54] Zhu J.K. (2002). Salt and drought stress signal transduction in plants. Ann. Rev. Plant. Biol..

[55] Orlowski J., Grinstein S. (2004). Diversity of the mammalian Na^+^/H^+^exchanger SLC9 gene family. Pflugers Arch..

[56] Bassil E., Blumwald E. (2014). The ins and outs of intracellular ion homeostasis: NHX-type cation/H^+^ transporters. Curr. Opin. Plant Biol..

[57] Blumwald E. (2000). Sodium transport and salt tolerance in plants. Curr. Opin. Cell Biol..

[58] Zhu J.K. (2003). Regulation of ion homeostasis under salt stress. Curr. Opin. Plant Biol..

[59] Zhang C., Hicks G.R., Raikhel N.V. (2013). Plant vacuole morphology and vacuolar trafficking. Front. Plant Sci..

[60] Moffat A.S. (2002). Plant genetics. Finding new ways to protect drought-stricken plants. Science.

[61] Brett C.L., Tukaye D.N., Mukherjee S., Rao R. (2005). The yeast endosomal Na^+^K^+^/H^+^ exchanger NHX1 regulates cellular pH to control vesicle trafficking. Mol. Biol. Cell.

[62] Chanroj S., Wang G., Venema K., Zhang M.W., Delwiche C.F., Sze H. (2012). Conserved and diversified gene families of monovalent Na^+^/H^+^ antiporters from algae to flowering plants. Front. Plant. Sci..

[63] Kinsella J., Aronson P. (1981). Amiloride inhibition of the Na^+^/H^+^ exchanger in renal microvillus membrane vesicles. Am. J. Physiol. Ren. Physiol..

[64] Blumwald E., Poole R.J. (1987). Salt tolerance in suspension cultures of sugar beet induction of Na^+^/H^+^ antiport activity at the tonoplast by growth in salt. Plant Physiol..

[65] Plesch G., Ehrhardt T., Mueller-Roeber B. (2001). Involvement of TAAAG elements suggests a role for Dof transcription factors in guard cell-specific gene expression. Plant J..

[66] Deniaud E., Baguet J., Chalard R., Blanquier B., Brinza L., Meunier J., Michallet M.C., Laugraud A., Ah-Soon C., Wierinckx A. (2009). Overexpression of transcription factor Sp1 leads to gene expression perturbations and cell cycle inhibition. PLoS ONE..

[67] Liu L., Xu W., Hu X., Liu H., Lin Y. (2016). W-box and G-box elements play important roles in early senescence of rice flag leaf. Sci. Rep..

[68] Bowers J.E., Chapman B.A., Rong J., Paterson A.H. (2003). Unravelling angiosperm genome evolution by phylogenetic analysis of chromosomal duplication events. Nature.

[69] Nagaraju M., Reddy P.S., Kumar A.S., Srivastava R.K., Kishor P.B.K., Rao D.M. (2015). Genome-wide scanning and characterization of *Sorghum bicolor* L. heat shock transcription factors. Curr. Genom..

[70] Ashnest J.R., Huynh D.L., Dragwidge J.M., Ford B.A., Gendall A.R. (2015). *Arabidopsis* intracellular NHX-type sodium-proton antiporters are required for seed storage protein processing. Plant. Cell. Physiol..

[71] Katiyar-Agarwal S., Zhu J., Kim K., Agarwal M., Fu X., Huang A., Zhu J. (2006). The plasma membrane Na^+^/H^+^ antiporter SOS1 interacts with RCD1 and functions in oxidative stress tolerance in *Arabidopsis*. Proc. Natl. Acad. Sci. USA.

[72] Quintero F.J., Martinez-Atienza J., Villalta I., Jiang X., Kim W.Y., Ali Z., Fujii H., Mendoza I., Yun D.-J., Zhu K.-K. (2011). Activation of the plasma membrane Na/H antiporter salt-overly-sensitive 1 (SOS1) by phosphorylation of an auto-inhibitory C-terminal domain. Proc. Natl. Acad. Sci. USA.

[73] Wu G., Wang G., Ji J., Tian X., Gao H., Zhao Q., Li J., Wang Y. (2014). Hydrophilic C terminus of *Salicornia europaea* vacuolar Na^+^*/*H^+^ antiporter is necessary for its function. J. Genet..

[74] Weinl S., Kudla J. (2009). The CBL-CIPK Ca^2+^-decoding signaling network: Function and perspectives. New Phytol..

[75] Liu J., Zhu J.K. (1998). A calcium sensor homolog required for plant salt tolerance. Science.

[76] Ishitani M., Liu J., Halfter U., Kim C.S., Shi W., Zhu J.K. (2000). SOS3 function in plant salt tolerance requires N-myristoylation and calcium binding. Plant Cell.

[77] Ma Y.C., Auge R.M., Dong C., Cheng Z.M.M. (2017). Increased salt tolerance with overexpression of Na^+^*/*H^+^antiporter 1 genes: A meta-analysis. Plant. Biotechnol. J..

