# Supplementary material for: Genome-Wide Identification and Analysis of Arabidopsis Sodium Proton Antiporter (NHX) and Human Sodium Proton Exchanger (NHE) Homologs in Sorghum bicolor
Source: Genes (Basel). 2018 May 3;9(5):236. doi: 10.3390/genes9050236 (PMC5977176; doi:10.3390/genes9050236)
Supplement: Supplementary file 1 [file genes-09-00236-s001.zip › Supplemental/Genes Figure S1, S2.pptx]

## Slide 1
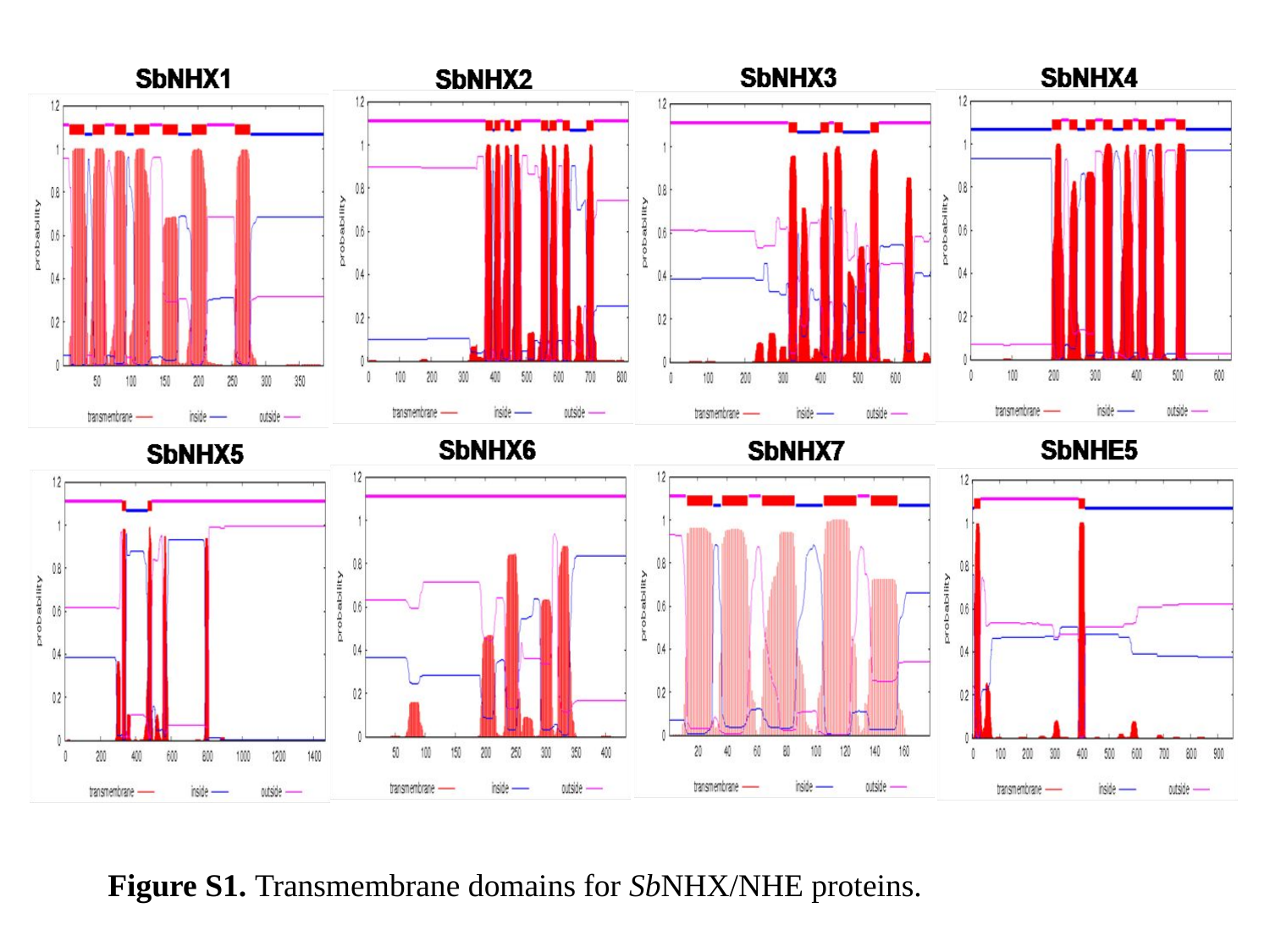

Figure S1. Transmembrane domains for SbNHX/NHE proteins.

## Slide 2
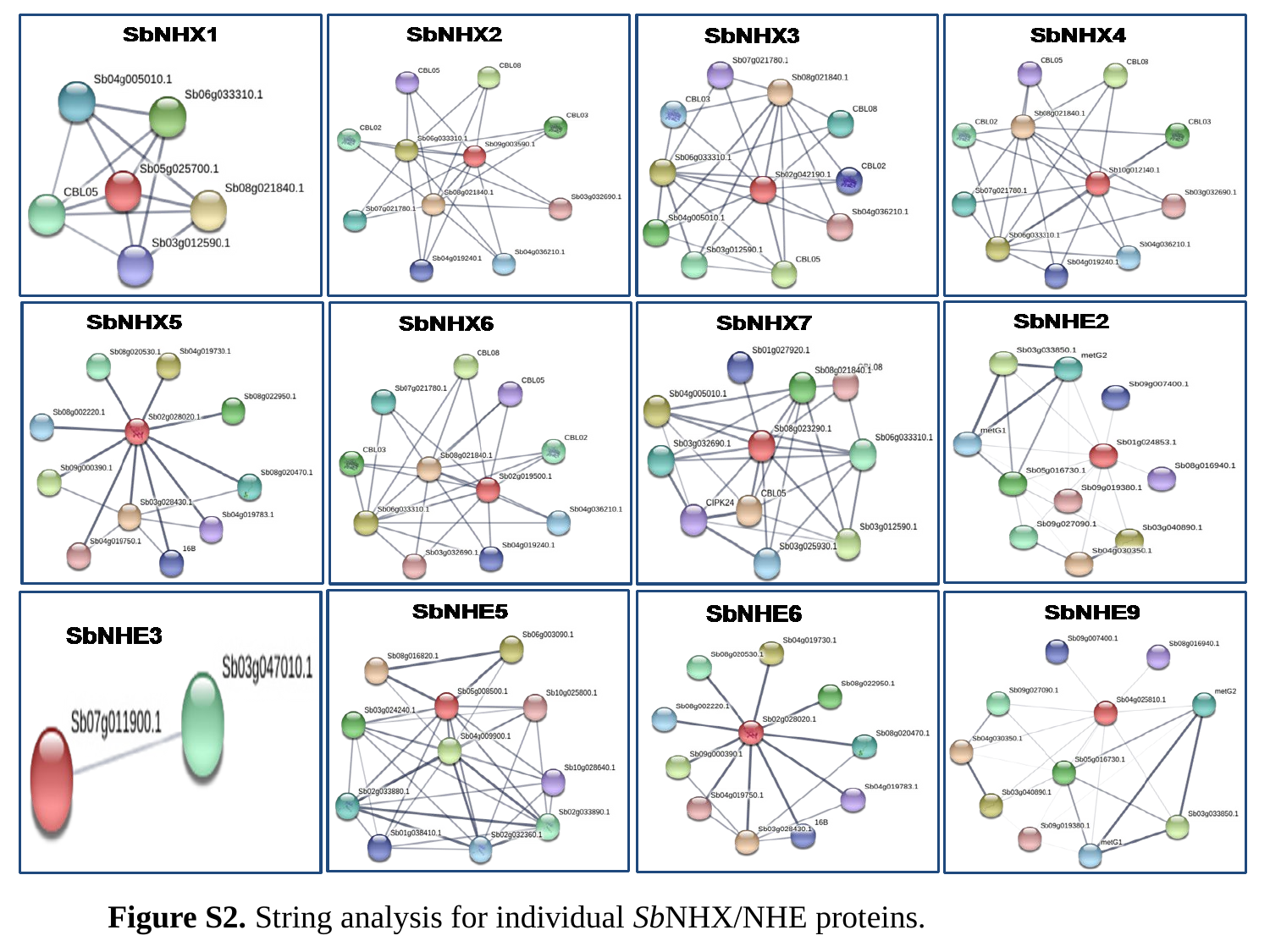

Figure S2. String analysis for individual SbNHX/NHE proteins.
